# The Healthcare Amyloidosis European Registry (HEAR): design of a national registry with a European extension strategy, and foundation of the F-CRIN GRACE network

**DOI:** 10.1186/s13023-025-04062-y

**Published:** 2025-10-27

**Authors:** Patricia Réant, Mounira Kharoubi, Erwan Donal, Fabrice Bauer, Mélanie Bézard, Arnaud Bisson, Diane Bodez, Océane Bouchot, Eve Cariou, Phillipe Charron, Jérôme Costa, Pierre-Yves Courand, Charlotte Dagrenat, Francois Delelis, Antoine Jobbe Duval, Jean-Christophe Eicher, Antoine Fraix, Barnabas Gellen, Jean-Pierre Gueffet, Damien Guijarro, Gilbert Habib, Albert Hagège, Olivier Huttin, Arnaud Jaccard, Julien Jeanneteau, Damien Legallois, Damien Logeart, Lise Legrand, Jocelyn Inamo, Léa Marguerit, Raphaël Mirailles, Théo Pezel, Nicolas Piriou, Francois Roubille, Basile Mouhat, Romain Trésorier, Jean-Jacques Von Hunolstein, Charles Taieb, Muriel Salvat, Amira Zaroui, Olivier Lairez, Thibaud Damy

**Affiliations:** 1 Bordeaux University Medical Center, Bordeaux University, CIC-P 1401, Bordeaux, France; 2 F-CRIN GRACE Network and Réseau Amylose, F-CRIN GRACE, 1 rue Gustave Eiffel, Creteil, F-94000 France; 3 Department of Cardiology, Referral Center for Cardiac Amyloidosis, Filiere Cardiogen, GRC Amyloid Research Institute, APHP CHU Henri Mondor, Créteil, France; 4 Heart’s Foundation, Vincennes, France; 5 Institut National de la Santé et de la Recherche Médicale (INSERM) U955, Clinical Epidemiology and Ageing (CEpiA) at Henri Mondor University Hospital and Institut Henri Mondor University Hospital, F-Créteil, France; 6 Université de Rennes, CHU Rennes, Service de Cardiologie Inserm, LTSI - UMR 1099, Rennes, France; 7 Department of Cardiology, CHRU Charles Nicolle, Rouen, France; 8 Department of Cardiology, CHRU de Tours, Tours, France; 9 Department of Cardiology, CHU Orleans, Orleans, France; 10 Department of Cardiology, Centre Cardiologique du Nord, Saint Denis, France; 11 Department of Cardiology, Centre Hospitalier Annecy Genevois, Epagny-Metz-Tessy, France; 12 Department of Cardiology, Cardiac Imaging Centre, University Hospital of Toulouse, Toulouse, France; 13 APHP, Department of Cardiology & Department of Genetics, Sorbonne Université, IHU ICAN, CHU Pitié Salpêtrière, Paris, France; 14 Department of Cardiology, CHU Robert Debré, Reims, France; 15 Department of Cardiology, Hôpital de la Croix Rousse et Lyon Sud, Hospice Civil de Lyon, Lyon, France; 16 Department of Cardiology, Centre Hospitalier Haguenau, Hagueneau, France; 17 Department of Cardiology, GHICL Hôpital St Vincent et St Philibert, Lille, France; 18 Department of Cardiology, Hôpital Louis Pradel, Bron, Hospice Civil de Lyon, Lyon, 69008 France; 19 Department of Cardiology, CHU de Dijon Bourgogne, Hôpital François Mitterrand, Dijon, France; 20 Department of Cardiology, CHRU de Nancy – Hôpitaux Brabois, Vandoeuvre lès Nancy, France; 21 Department of Cardiology, Polyclinique de Poitiers Elsan, Poitiers, France; 22 Department of Cardiology, Hôpital Privé Confluent, Nantes, France; 23 Department of Cardiology, Groupe Hospitalier Mutualiste, Grenoble, France; 24 Department of Cardiology, Assistance Publique Hôpitaux de Marseille, CHU de La Timone, Marseille, France; 25 Department of Cardiology, APHP Centre Hospitalier Georges Pompidou, Paris, France; 26 Faculty of Medicine, Paris Descartes University, Sorbonne Paris Cité, Paris, France; 27 Paris Cardiovascular Research Center, INSERM, UMR-970, Paris, France; 28 Department of Cardiology, CHU Dupuytren, Limoges, France; 29 Department of Cardiology, Clinique Saint Joseph, Trelazé, France; 30 Department of Cardiology, CHU de Caen, Caen, France; 31 Department of Cardiology, APHP, CHU Lariboisière, Paris, France; 32 Department of Cardiology, APHP CHU Pitié Salpétrière, Paris, France; 33 Department of Cardiology, CHU de Martinique, Fort de France, France; 34 MIRACL.ai Laboratory, Multimodality Imaging for Research and Analysis Core Laboratory and Artificial Intelligence, Lariboisiere University Hospital (AP-HP), Paris, France; 35 Nantes Université, CHU Nantes, INSERM, Cardiology Department, CIC 1413, Institut du Thorax, Nantes, France; 36 Department of Cardiology, CHU de Montpellier, Montpellier, France; 37 Department of Cardiology, CHU de Besancon, Besancon Cedex, France; 38 Department of Cardiology, CHU de Clermont Ferrand, Clermont Ferrand, France; 39 Department of Cardiology, Nouvel Hôpital Civil – Hôpitaux Universitaires de Strasbourg, Strasbourg, France; 40 European Market Maintenance Assessment, Fontenay sous-Bois, France; 41 Department of Cardiology, CHU Grenoble-Alpes, La Tronche, France

**Keywords:** Registry, F-CRIN GRACE network, Cardiac amyloidosis, Outcomes, Quality of life, Treatment

## Abstract

**Background:**

Cardiac amyloidosis (CA) is a rare disease that can lead to poor quality of life, conduction disorders, arrhythmia, heart failure, and even death. Fortunately, specific treatments that can modify the natural history of the disease and the disease outcomes are now available. However, data on the prevailing patient management procedures and long-term outcomes of CA are scarce.

**Objective:**

The Healthcare Amyloidosis European Registry (HEAR) is 34-centre registry initiated in France and structured for European expansion through the French Clinical Research Infrastructure Network’s Group for Research on Amyloidosis and Care Excellence and the European Clinical Research Infrastructure Network. We expect to include 8500 patients between January 2021 and December 2027.

**Methods:**

The HEAR has been designed to capture detailed demographic, clinical, laboratory, imaging, and therapeutic data from both suspected and confirmed cases of all cardiac amyloidosis subtypes, including wildtype transthyretin amyloidosis, variant transthyretin amyloidosis, light-chain amyloidosis, and rarer forms. This comprehensive approach has been designed to (i) improve our understanding of real-world diagnostic pathways, treatment practices, and patient outcomes and (ii) incorporate patient-centred innovations. To enhance the patient-centred nature of the registry, patient-reported outcome measures (PROMs) will be systematically collected.

**Conclusions:**

By addressing diagnostic pathways, real-world management and PROMs and by applying technological innovations and European scalability, the next-generation HEAR is establishing itself as a valuable resource for clinical research, public health interventions, and better patient care in the field of CA.

**Supplementary information:**

The online version contains supplementary material available at 10.1186/s13023-025-04062-y.

## Key summary points

The Healthcare Amyloidosis European Registry is a longitudinal, multicentre registry of patients referred for suspected or confirmed cardiac amyloidosis.

Since July 2021, 34 hospitals across France have joined the Healthcare Amyloidosis European Registry. The registry has been designed for extension to other European countries, thanks to its accreditation as the French Clinical Research Infrastructure Network’s Group for Research on Amyloidosis and Care Excellence.

**Supplementary information:**

The online version contains supplementary material available at 10.1186/s13023-025-04062-y.

## Introduction

Cardiac amyloidosis (CA) is a rare disease characterized by the continuous accumulation of infiltrating, insoluble fibrillar proteins in the extracellular matrix of various organs, including the kidneys, nerves, liver, heart, and skeletal muscles [1]. The prevalence of CA in Europe is not known [2]; however, data from the Transthyretin Amyloidosis Outcomes Survey (THAOS) suggest that the prevalence of wild-type (non-hereditary) TTR amyloidosis (ATTRwt) in North America and Europe ranges from 13 to 18 cases per million [3]. Cardiac involvement is frequently observed for three types of systemic amyloidosis: (i) amyloid light chain amyloidosis (caused by excess monoclonal light chain production by a plasma cell clone), (ii) hereditary transthyretin (TTR) amyloidosis (ATTRv, inherited in an autosomal dominant manner and caused by the deposition of mutant TTR protein), and (iii) wild-type (non-hereditary) TTR amyloidosis (ATTRwt).

For a patient with amyloidosis, the prognosis typically depends on the state of cardiac involvement [4, 5]; unfortunately, CA is often diagnosed late in the course of the disease, when the prognosis is poor. ATTRwt mainly affects men over the age of 60, whereas ATTRv affects both women and men and usually appears between the ages of 40 and 60 (depending on the mutation). This diagnostic delay is due to several factors: (i) the level of amyloid infiltration needed to generate symptoms; (ii) the amyloid fibrils’ speed of accumulation; (iii) a low level of awareness of CA among physicians; and (v) the lack of simple diagnostic tools (biomarkers, imaging, etc.) for early diagnosis. The absence of early diagnosis, the heterogeneity of the multisystem expression of amyloidosis, and the complexity of patient management result in diagnostic delays, failure to treat organ damage, and degradation of the patient’s quality of life (QoL) and chances of survival [4, 6–8].

Hence, physicians must better recognize the cardiac and extracardiac signs of amyloidosis, characterize the disease’s clinical and biochemical presentations and their times of onset, improve diagnostic tools, and adopt standardized patient management procedures. International and national registries and observatories [8] are important tools for improving the quality of care [3, 6, 9–13]. These tools make it possible to spread information to the entire community with regard to care practices and the latter’s changes over time and impacts on public health. This is particularly true in the field of cardiology. Thus, it has been demonstrated that the countries that implemented national registers a long time ago (such as Sweden) have improved their practices more quickly than countries that took this step later (such as the United Kingdom) [14].

Several national and international registries have been developed, in order to better characterize CA and related conditions. Among the most prominent are the THAOS [3, 13], the Systemic Amyloidosis in Europe (EURAMY) network [15], the UK-based National Amyloidosis Centre [16], all of which have significantly improved the understanding of the disease’s natural history, genotype-phenotype correlations, and therapeutic outcomes. When compared with existing registries, the Healthcare European Amyloidosis Registry (HEAR) presented here constitutes an innovative, comprehensive approach (Table 1).

**Table 1:** Comparison of the HEAR with existing cardiac amyloidosis registries

Registry	THAOS	NAC Database	EURAMY	HEAR
Amyloidosis subtypes included	ATTR-only	All subtypes (ATTR, AL, others)	AL mainly	All subtypes (ATTR, AL, others)
Suspected cases included	No	No	No	Yes
PROMs collected	Optional/inconsistent	?	No	Yes (AmyloAFFECT-QOL, MLHFQ, KCCQ)
Centralized imaging	No	No	No	Yes (AI-based via MIRACL.ai)
PREMs collected	No	No	No	Planned (AI patient chatbot)
Linkage to national databases	No	No	No	Planned
Linkage to clinical research and trial networks	No	?	No	GRACE F-CRIN/E-CRIN
Governance	Industry-sponsored	Single-centre academic	Centre-dependent	Multicentre academic
Scalability and future strategy	Terminated	Will include several centres in UK	Terminated	European expansion

THAOS: Transthyretin Amyloidosis Outcomes Survey; NAC: National Amyloidosis Centre; EURAMY: Systemic Amyloidoses in Europe; HEAR: Healthcare Amyloidosis European Registry; ATTR: Transthyretin amyloidosis; AL: Light chain amyloidosis; PROM: MLHFQ: Minnesota Living Heart Failure Questionnaire; KCCQ: Kansas City Cardiomyopathy Questionnaire; PREM: patient-reported experience measures; MIRACL.ai: Multimodality Imaging for Research and Analysis Core Laboratory and Artificial Intelligence; AI: Artificial Intelligence; GRACE: Group for Research on Amyloidosis and Care Excellence: F-CRIN: French Clinical Research Infrastructure Network; E-CRIN: European Clinical Research Infrastructure Network

Existing registries differ in scope, geographic coverage, inclusion criteria, and long-term follow-up strategies. Furthermore, most existing datasets are limited to specific clinical settings, national frameworks, or pharmaceutical-sponsored initiatives; this potentially creates gaps in harmonized data collection across Europe. To address this need, the HEAR was initiated as an independent registry with scope for expansion across Europe. The goal is to provide a unified, collaborative platform for the collection of standardized clinical, genetic, and treatment-related data across multiple countries. This should support research, care optimization, and policy-making in the field of amyloidosis, accelerate improvements in practice, and thus extend survival times for patients [14].

In order to bridge this knowledge gap and collect representative data, we have initiated the Healthcare Amyloidosis European Registry (HEAR) in France and are planning to extend it to other European countries. The HEAR is sponsored by Heart’s Foundation (Vincennes, France) and is already Europe’s largest multicentre registry of CA. Its primary objective is to characterize the natural history of CA by capturing detailed clinical and diagnostic data at the time of diagnosis, documenting current treatment practices, and assessing patient outcomes in real-world settings. Building on this foundation, collaborative efforts seek to advance the understanding of this rare disease, generate critical evidence, and thus inform future guidelines for optimal diagnosis and management. The present report outlines the design and implementation of the HEAR, which has been developed in France as the new, operational platform for the French Clinical Research Infrastructure Network (F-CRIN)’s Group for Research on Amyloidosis and Care Excellence (GRACE). The F-CRIN https://www.fcrin.org/en) supports research, expertise and clinical investigation networks in targeted medical fields linked to the European Clinical Research Infrastructure Network (E-CRIN, https://ecrin.org/). Through the GRACE, the F-CRIN and the E-CRIN, the HEAR will benefit from expertise in harmonizing data collection tools, support in regulatory alignment across European countries, and access to a network of national scientific coordinators (clinical trial units and research infrastructures) across Europe.

## Methods

### Objectives of the registry

The HEARs primary objective is to describe the demographic, clinical, laboratory data and imaging characteristics and the QoL of patients referred for suspected CA and patients with a confirmed diagnosis of CA. It will thus provide insights into real-world diagnosis pathways.

The registry’s secondary objectives are to (i) estimate the prevalence and incidence of the different types of amyloidosis and their changes over time, (ii) describe the cardiac and extracardiac cardiac signs and their time of appearance, depending on the diagnosis, (iii) describe the course of care (diagnosis pathway and time) and its change over time, (iv) assess the quality of diagnostic tools (biomarkers, imaging, etc.) and algorithm diagnosis [17], (v) describe patient QoL and well-being, using the AmyloAFFECT-QOL [7] (a validated, amyloidosis-specific questionnaire), the Minnesota Living Heart Failure Questionnaire (MLHFQ), and the Kansas City Cardiomyopathy Questionnaire (KCCQ), (vi) describe the disease prognosis and its change over time, (vii) describe cardiological and specific treatments and their benefits and side effects, (viii) assess the number and reasons for hospital admissions and medical and surgical interventions, (ix) link to other databases (e.g. health insurance databases), and (x) share good practice and thus evaluate and optimize the quality of healthcare for patients with CA in France.

### Design and sampling

The HEAR is a French, multicentre, observational registry for patients with suspected or confirmed CA. We expect to include 8,500 patients between July 2021 and December 2027. The registry will comprise retrospective, retroprospective and prospective cohorts.

Firstly, the HEAR RETROSPECTIVE cohort will comprise deceased patients with confirmed diagnosis of CA, who met the inclusion criteria and were treated at the centre after 2009. The data from this cohort will enable us to characterize the natural history of the disease.

Secondly, the HEAR RETROPROSPECTIVE cohort will comprise live patients with a confirmed diagnosis of CA and real-life follow-up from the date of inclusion, who have been treated after 2009 and are still being monitored at the centre. The patients will be asked to give their written, informed consent for the retroprospective and prospective collection of their personal data.

Thirdly, the HEAR PROSPECTIVE cohort will comprise patients referred to the participating centres for suspected CA. The real-life data from this cohort will enable us to characterize (i) diagnostic pathways for patients with suspected amyloidosis and (ii) changes in the management of CA. The patients will be asked to give their written, informed consent for the prospective collection of their personal data.

We are collecting demographic, clinical, laboratory, imaging, and QoL data (via the systematic completion of the AmyloAFFECT-QOL questionnaire, the MLHFQ and the KCCQ) and diagnostic data at baseline for all registered patients, by using a structured electronic case report form (e-CRF)). We will also collect data on patient management by the cardiologists and specific details of treatments. Lastly, we intend to collect in-hospital data on outcomes (deaths, cause of death, and hospital readmissions) annually.

Furthermore, the HEAR will include a digital health component through the development of an artificial-intelligence-powered patient chatbot designed to (i) provide educational support and personalized guidance, (ii) explain quality-of-life questionnaires in accessible terms, (iii) facilitate the completion of patient-reported outcome measures (PROMs), (iv) collect patient-reported experience measures), and (v) promote health literacy and patient empowerment.

### Participating centres

**Fig. 1:**
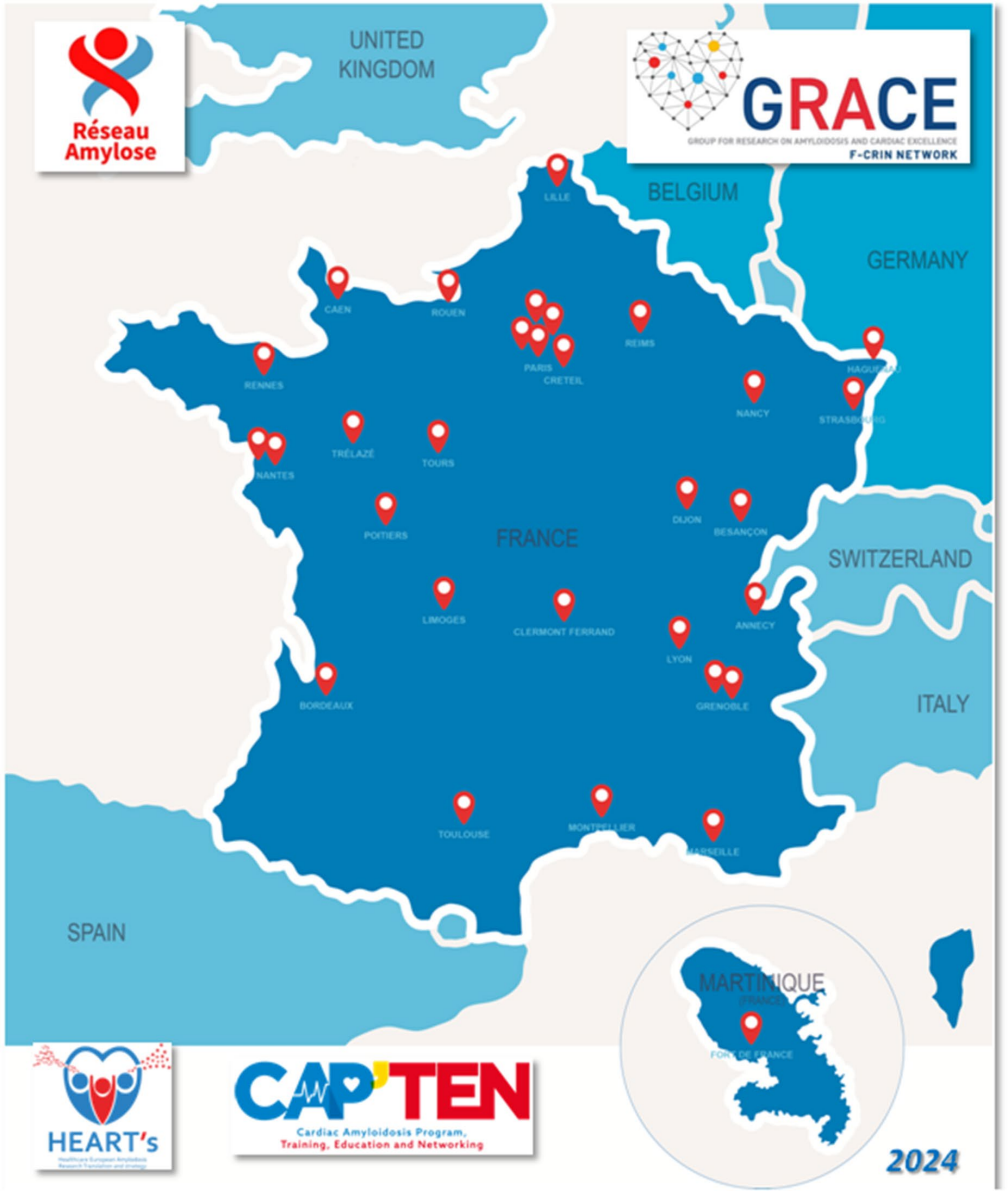
A map showing the participating centres in France

**Fig. 2:**
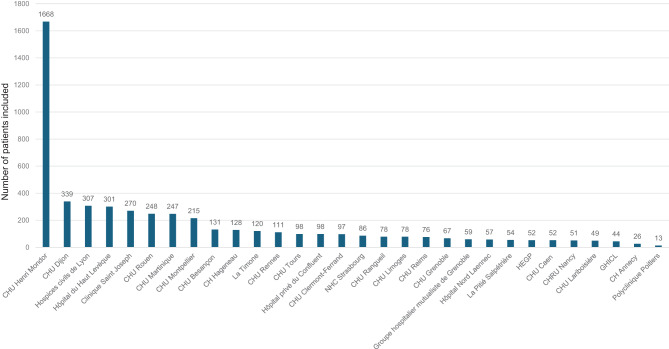
The numbers of patients included at each centre (as of March 1^st^, 2024)

The lead investigator (an experienced researcher and a cardiologist at the French National Cardiac Amyloidosis Reference Centre) identified 34 hospitals and invited them to participate in the HEAR; all accepted (Appendix A and Fig. 1). The 34 participating centres are representative of France’s geographic and ethnic diversity. Each centre has been asked to register consecutive patients. The distribution of patient inclusions by centre as of March 1^st^, 2024, is shown in Fig. 2. Four centres had not started recruitment at that date. Lastly, it should be noted that we intend to extend the HEAR to other European countries.

### Definition, inclusion, and exclusion criteria

We apply the diagnostic criteria for CA given in the European Society of Cardiology’s 2021 expert consensus [17]. The criteria for patient inclusion in the HEAR are as follows: age 18 or over, referral for suspected CA or a confirmed diagnosis of CA, and coverage by the French social security system. The main exclusion criterion was refusal to participate in the registry. Patients included in the HEAR do not receive any financial compensation.

### Study tools

Clinical data are collected using an e-CRF that can be accessed over the Internet by the participating physicians and the clinical study coordinator. Data related to the centre and the details of each participating physician (family name, first name, postal address, e-mail addresses, telephone number, fax numbers, age, sex, medical specialty, and department) are collected via another e-CRF.

The patient-related data collected for the HEAR include demographic variables (date of birth, sex, country of origin, and lifestyle) and medical variables (medical history, symptoms, clinical, laboratory and imaging data, treatments, and genetic test results for cardiomyopathies).

### Longitudinal follow-up

Given that the study is observational, patients will be followed up according to each centre’s standard procedures; the patient’s management will not be modified and no additional visits will be required. However, vital status and clinically significant events will be collected for all patients included in the HEAR RETROSPECTIVE cohort. In the absence of a visit to the centre or inclusion in the HEAR PROSPECTIVE cohort (see below), the vital status (alive or dead) of patients will be confirmed successively by (i) consulting the centre’s appointment scheduling software, (ii) phoning the patient or a relative if the patient had not attended the participating centre after the study set-up, (iii) contacting the patient’s general practitioner by phone or by post, and (iv) contacting the town hall corresponding to the patient’s last known address by phone or by post.

For all patients included in the HEAR PROSPECTIVE cohort, vital status (alive or dead) will be checked annually. If a year goes by without the collection of data (i.e. failure to visit the study centre), the investigators will seek to document vital status and events that occurred in the previous year by carrying out the procedures described above for the HEAR RETROSPECTIVE cohort.

In accordance with the current French legislation and regulations, adverse events (whether serious or not) will be notified to the competent authorities by the study investigators. These events will be recorded by the investigators throughout the study, using the dedicated sections of the e-CRF.

### Collection of the data

The data will be entered in the e-CRF directly by the investigating physicians. Consistency tests will be run on the study database, in order to check the consistency of dates (given the timeline of events) and the most important major variables. These checks will be defined in a data validation plan validated by the study sponsor (Heart’s Foundation).

The data’s completeness and quality and the level of compliance with study procedures will be checked regularly throughout the study.

Patient inclusions and status will be monitored on a regular basis by the study’s clinical research associates. Investigators will be invited to contact any patients lost to follow-up.

The principal investigator will use a specific remote access to check that the e-CRF is completed correctly with regard to data completeness and quality. The clinical study coordinator will also be in charge of sending the participating physicians data queries generated by the consistency tests and ensuring that the queries are answered in full. To this end, regular telephone interviews between the clinical study coordinator and the investigators will be help the latter to fill in and correct (if required) the data.

The investigating physicians have authorized the sponsor or any person authorized by the sponsor to carry out on-site audits (during or at the end of the study), in order to check that the study is being conducted in accordance with the approved protocol. The competent authorities may order an inspection. The investigators must guarantee the quality and authenticity of the data collected and compliance with ethical principles.

### Data collection category

The comprehensive set of variables collected within the HEAR database have been structured to ensure robust, standardized, and longitudinal data collection aligned with international clinical research standards (Table 2). Each variable falls under a defined data category and is associated with specific time points of collection (such as screening, follow-up, or both), in order to capture the dynamic nature of disease progression, therapeutic intervention, and patient outcomes. The registry includes critical domains such as patient demographics, the amyloidosis subtype, genetic characterization, detailed general and cardiological medical histories, specific symptoms related to amyloidosis, and therapeutic regimens. Furthermore, the registry includes vital signs, biomarker data, multimodal cardiac imaging (e.g., echocardiography, MRI, and scintigraphy), biopsy findings, and adverse event reports. Importantly, patient-reported outcomes (e.g. QoL) are collected as a guide to the impact of the disease and treatments on daily living. This structured, exhaustive data framework is essential for enabling longitudinal analyses, fostering translational research, and supporting real-world evidence generation in the field of CA.

**Table 2:** Variables collected in the HEAR database

Data category	Examples of collected variables	Time of collection	Comments
Informed consent	Date, version of consent form	Screening	ICH-GCP compliant
Demographics	Age, sex, weight, height	Screening	Baseline characteristics
Cardiomyopathy	Cardiac amyloidosis (ATTRv, ATTRwt, AL), ATTRv asymptomatic, AA, ApoA1, ApoA2, ApoA3, ApoA4, Other cardiomyopathy	Screening	
Type of mutation	Cardiac genotype, neurologic genotype, mixed genetic	Screening	
General medical history	Family history, allergies, comorbidities (AHT, diabetes, dyslipidaemia)	Screening	Baseline characteristics
Cardiac medical history	Heart failure, Cardiac Valvular disease, Cardiac Arrhythmia, Cardiac Device	Screening	Baseline characteristics
Amyloidosis-specific medication	ATTR (tafamidis, patisiran, vutrisiran, and inotersen) and AL (chemotherapy): dosing regimen, start date, stop date	Screening + follow-up	
Concomitant medication	Drug name, dosing regimen, duration	Screening + follow-up	Potential interaction with treatment
Symptoms	First cardiac and extracardiac symptoms, deafness, orthostatic hypotension, dysautonomia, carpal tunnel syndrome, neuropathy, Dupuytren contracture, lumbar canal, hip prosthesis, knee prosthesis	Screening + follow-up	
Vital signs	NYHA, Blood pressure, heart rate	Screening + follow-up visits	
Laboratory data	NT-proBNP, BNP, troponin T, troponin I creatinine, GFR, liver enzyme, albumin, prealbumin, lipid panel, iron panel, interleukin-6, calprotectin (MRP8/14),	Screening + follow-up visits	
Electrocardiogram data	Heart rate, rhythm, QRS interval, PR interval	Screening + follow-up visits	
Echocardiography data	IVS, LVEF, LV global strain, TAPSE, PASP, E/A, E/E’	Screening + follow-up visits	
Tissue biopsy	Salivary gland, cardiac biopsy, carpal tunnel biopsy, nerve biopsy,	Screening + follow-up (if needed)	
MRI	LVEF, T1, T2, ECV	Screening + follow-up (if needed)	
Scintigraphy	HMDP/DPD/PYP. Late phase visual scoring, H/CL lung ratio. Early phase: heart-to-mediastinum ratio	Screening + follow-up (if needed)	
Medication	All treatments and changes	Baseline and follow-up	Amyloidosis treatment and others
Cardiac adverse events and vital status and cause of death	Type, severity, relationship with treatment (heart failure, valve failure, thromboembolic event, coronary event, conduction event, arrhythmia event, other cardiac event	Follow-up	Reported in accordance with GCP
Extracardiac adverse events	Infectious event, dialysis, haemorrhagic event, other extracardiac event.	Follow-up	
Quality of life questionnaire	AmyloAFFECT-QoL, MLHFQ, KCCQ	Screening + follow-up	

ICH-GCP: International Council for Harmonisation - Good Clinical Practice; ATTRv: Transthyretin amyloidosis, variant (hereditary); ATTRwt: Transthyretin amyloidosis, wild-type; AL: Light chain amyloidosis; AA: Serum amyloid A amyloidosis; Apo: Apoprotein; AHT: Arterial hypertension;, NYHA: New York Heart Association; NT-proBNP: N-terminal B-type natriuretic peptide; BNP: B-type natriuretic peptide; GFR: Glomerular filtration rate; IVS: Interventricular septum; LVEF: Left ventricular ejection fraction; TAPSE: Tricuspid annular plane systolic excursion; PASP: Pulmonary artery systolic pressure; ECV: Extracellular volume; HMDP: Hydroxymethylene diphosphonate; DPD: Diphosphonopropanodicarboxylic acid; PYP: Pyrophosphate; HCL: Heart-to-contralateral-lung; QoL: Quality of life; MLHFQ: Minnesota Living Heart Failure Questionnaire; KCCQ: Kansas City Cardiomyopathy Questionnaire

### Core laboratory for cardiovascular imaging

To enhance the robustness of the study for cardiovascular imaging findings, several participating centres will used specialized software to send pseudo-anonymized DICOM images to central labs for analysis. An innovative aspect of the HEAR is centralized MRI using artificial intelligence algorithms managed by the Multimodality Imaging for Research and Analysis Core Laboratory and Artificial Intelligence (MIRACL.ai) at Lariboisière University Hospital (Paris, France). This process is supervised by experts with European Association of Cardiovascular Imaging Level III certification. The MIRACL.ai will comprehensively assess each examination (including inter- and intra-observer reproducibility) and apply various post-processing techniques (such as strain analysis and artificial intelligence algorithms).

### Ethical considerations

The study will be conducted and reporting on in accordance with the principles of the Declaration of Helsinki. The HEAR is registered with the French National Agency for the Safety of Medicines and Health (*Agence nationale de sécurité du médicament et des produits de santé* (Paris, France); identifier: IDRCB 2019-A02010-57) and ClinicalTrials.gov (identifier: NCT05101304, registered on 2021–10-19). As stated above, the HEAR protocol is strictly observational. It will not modify the patient’s usual care and will not require additional consultations or examinations.

## Discussion

**Fig. 3:**
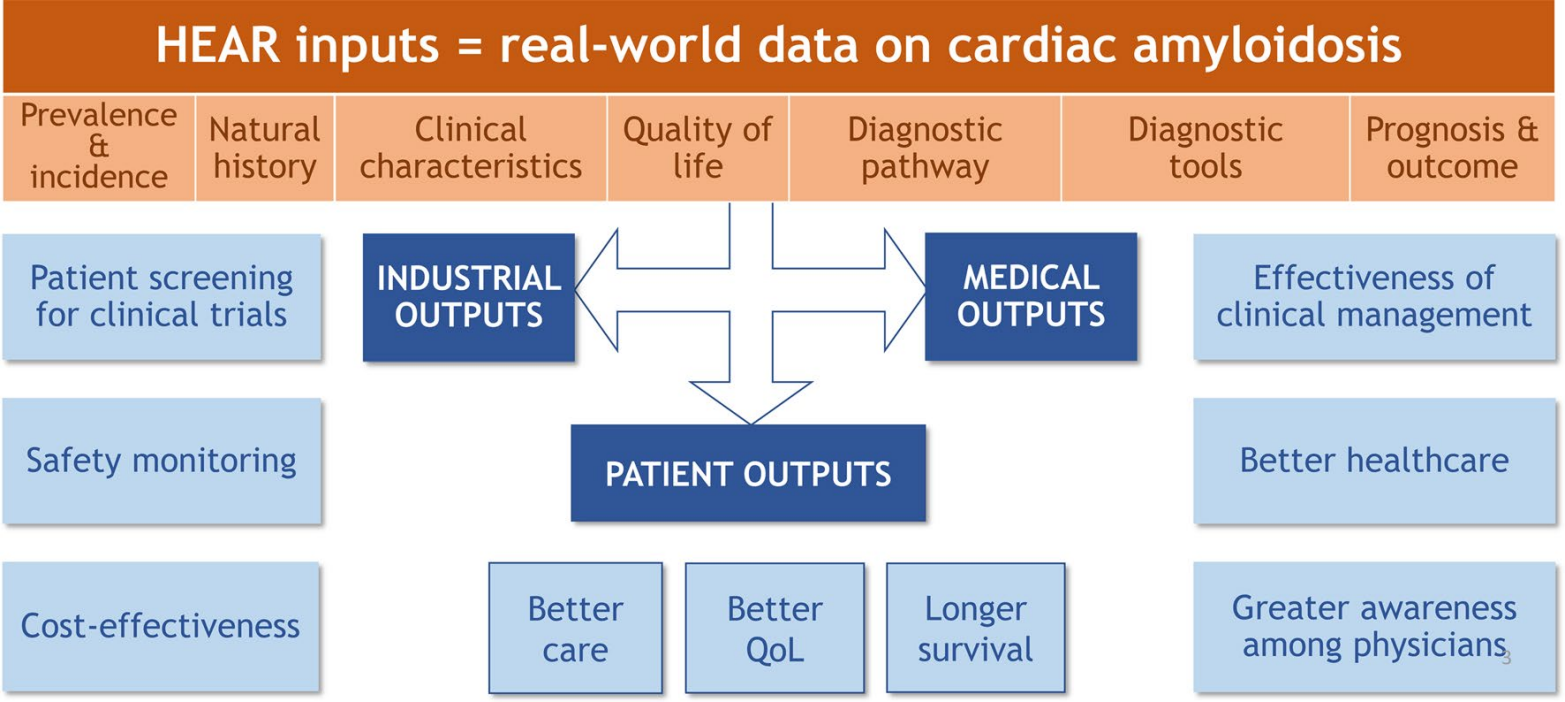
The HEAR's inputs and outputs for patients, physicians, and industrial partners

The European Society of Cardiology’s recently published guidelines emphasize the importance of collaboration between centres and the establishment of networks, so that patients can be referred to regional or national centres for complex diagnostic procedures and decision-making [17]. As the number of patients with CA continues to grow, we have set up the HEAR with a view to combating misdiagnosis, generating scientific and medical knowledge, improving patient care, and evaluating tools for diagnosis and for the assessment of QoL. The HEAR will also help to raise awareness of CA among healthcare professionals and to improve the care of patients with CA (especially those diagnosed recently). This multidisciplinary effort constitutes a unique opportunity to define early diagnostic findings, describe the natural history of CA, monitor patient outcomes, and improve our overall understanding of ATTR. The HEARs inputs and outputs for patients, physician and industrial partners are summarized in Fig. 3. For instance, HEAR allowed the comparison of survival outcomes in patients aged ≥80 years with cardiac transthyretin amyloidosis before and after the availability of tafamidis [18], which underscores the registry’s value as a real-world data platform for evaluating therapeutic effectiveness, patient trajectories, and healthcare strategies in CA.

The HEAR will also foster the implementation of clinical trials by bringing together research centres and facilitating patient screening with regard to clinical trial inclusion and exclusion criteria.

The guidelines recently published by the European Society of Cardiology emphasize the critical importance of collaboration between specialized centres and the development of structured care networks, allowing patients to be referred to regional or national hubs for complex diagnostic procedures and expert multidisciplinary decision-making [17]. In response to the growing number of patients diagnosed with CA, the HEAR was initiated with the objective of addressing diagnostic delays, generating robust scientific and medical data, improving care pathways, and evaluating both diagnostic tools and patient-reported outcomes, including QoL.

Beyond clinical data collection, the HEAR also seeks to raise awareness of CA among healthcare professionals, especially in the context of early or recent diagnoses, and to strengthen harmonized care approaches. This multidisciplinary, collaborative platform offers a unique opportunity to define early diagnostic features, document the natural history of hereditary forms of amyloidosis, monitor patient outcomes longitudinally, and deepen our understanding of transthyretin amyloidosis (ATTRv) in real-world settings.

Importantly, the HEAR in F-CRIN is also positioned to support clinical research and trial readiness by streamlining patient identification according to trial-specific inclusion and exclusion criteria and by fostering synergies across participating academic and clinical centres. As part of our strategy to expand across Europe, we intend to translate the eCRF into other European languages, harmonize data collection with ESC guidelines, deploy it through E-CRIN networks, and thus leveraging the latter’s operational and legal expertise.

With this framework, the integration of the HEAR into established research infrastructures such as F-CRIN and ECRIN is essential for maximizing the registry’s scientific and operational impact. F-CRIN provides nationwide support for excellence in clinical research in France, including methodological expertise, trial management, and regulatory guidance. ECRIN will enables Europe-wide coordination, harmonization of standards, and logistic facilitation of multinational trials. Together, these platforms enhance the credibility, sustainability and scalability of the HEAR as a reference registry in Europe, while reinforcing the latter’s ability to generate high-quality data, empower clinical trials, and ultimately improve outcomes for patients with CA.

## Conclusions

The HEAR will include a large number of patients with suspected or confirmed CA and will provide an opportunity to collect a large amount of data, better understand the disease and care pathways, and facilitate the inclusion of patients in clinical trials.

## Electronic supplementary material

Below is the link to the electronic supplementary material.

Supplementary Material 1: Appendix A: A list of the participating centres and investigators.

## Data Availability

Not applicable.

## Abbreviations

AA: Serum amyloid A amyloidosis
AHT: Arterial hypertension
AL: Light chain amyloidosis
Apo: Apoprotein
ATTR: Transthyretin amyloidosis
ATTRv: Transthyretin amyloidosis, variant (hereditary)
ATTRwt: Transthyretin amyloidosis, wild-type
BNP: B-type natriuretic peptide
CA: Cardiac amyloidosis
DPD: Diphosphonopropanodicarboxylic acid
e-CRF: Electronic case report form
F-CRIN: French Clinical Research Infrastructure Network
E-CRIN: European Clinical Research Infrastructure Network
ECV: Extracellular volume
GFR: Glomerular filtration rate
GRACE: Group for Research on Amyloidosis and Care Excellence
HCL: Heart-to-contralateral-lung
HEAR: Healthcare Amyloidosis European Registry
HMDP: Hydroxymethylene diphosphonate
ICH-GCP: International Council for Harmonisation - Good Clinical Practice
IVS: Interventricular septum
KCCQ: Kansas City Cardiomyopathy Questionnaire
LVEF: Left ventricular ejection fraction
MLHFQ: Minnesota Living Heart Failure Questionnaire
NT-proBNP: N-terminal B-type natriuretic peptide
NYHA: New York Heart Association
PASP: Pulmonary artery systolic pressure
PREM: patient-reported experience measure
PROM: patient-reported outcome measure
PYP: Pyrophosphate
QoL: Quality of life
TAPSE: Tricuspid annular plane systolic excursion
THAOS: Transthyretin Amyloidosis Outcomes Survey
TTR: Transthyretin
v: Variant (hereditary)
wt: Wild-type
